# Chitosan complements entrapment of silicon inside nitrogen doped carbon to improve and stabilize the capacity of Li-ion batteries

**DOI:** 10.1038/s41598-019-39988-4

**Published:** 2019-03-01

**Authors:** K. Prasanna, T. Subburaj, Yong Nam Jo, P. Santhoshkumar, S. K. S. Saravana Karthikeyan, Kumaran Vediappan, R. M. Gnanamuthu, Chang Woo Lee

**Affiliations:** 1 Department of Energy Conversion and Storage, Technical University of Denmark, Frederiksborgvej 399, P.O. Box 49, DK-4000 Roskilde, Denmark; 2 Department of Chemical Engineering, National Taiwan University Taipei, Taiwan, Republic of China; 3 Department of Advanced Materials & Chemical Engineering, College of Engineering, Halla University, 28 Halladaegil, Wonju, Gangwon 26404 South Korea; 4 Department of Chemical Engineering, Center for the SMART Energy Platform, College of Engineering, Kyung Hee University, 1732 Deogyeong-daero, Gihung, Yongin, Gyeonggi 17104 South Korea; 5 SRM Research Institute and Department of Chemistry, SRM Institute of Science and Technology, Kattankulathur, Chennai, Tamilnadu 603203 India

## Abstract

A facile strategy to entrap milled silicon (m-Si) particles using nitrogen-doped-carbon (N-C@m-Si) to overcome the dramatic volume changes in Si during intercalation of lithium ions and to improve its electronic conductivity is reported here. The only natural nitrogen containing biomaterial alkaline polysaccharide, i.e., chitosan, is used as the carbon source. Simple hydrothermal technique followed by a subsequent carbonization process is used to synthesize N-C and N-C@m-Si particles. N-C@m-Si exhibited significantly improved electrochemical performance as compared to bare m-Si, which is confirmed by the obtained discharge capacity of 942.4 mAh g^−1^ and columbic efficiency of 97% after 50 cycles at 0.1C rate. With regard to the N-C electrodes, the obtained discharge capacity of 485.34 mAh g^−1^ and columbic efficiency of 99.78%, after 50 cycles at 0.1C rate is superior to the commercial graphite electrodes. The solid electrolyte interphase (SEI) layer that formed over m-Si and N-C@m-Si electrodes is characterized using X-ray photoelectron spectroscopy. Compared to the SEI layer that formed over m-Si electrode after 10 charge-discharge cycles, the N-C@m-Si electrode had a stable lithium fluoride and carbonate species. Brief reaction mechanisms, representing the formation of different species in the SEI layer, is derived to explain its behavior during the electrochemical processes.

## Introduction

Current Li-ion batteries support extended usage of portable electronic devices and electric vehicles before charging is required. Nevertheless, there is a great demand for Li-ion batteries that can hold more power and provide higher energy than the existing ones. This has led to an increased research into the materials used during Li-ion battery fabrication. For practical applications, low-cost materials with high efficiency are required. Another important consideration is the large-scale production capability at a low price^1^.

With regard to the anode materials used in Li-ion batteries, graphite plays a dominant role due to its low volume expansion, high reversibility, and stable capacity with prolonged cycling. However, it has a major drawback of low theoretical capacity (≈372 mAh g^−1^)^2,3^. Researchers have recently shifted their focus to high theoretical capacity anode materials such as tin (≈994 mAh g^−1^)^4^ and silicon (≈4200 mAh g^−1^)^5,6^. Si is considered as a promising material for future devices because of its attractive voltage profile, abundance, low cost, and environmental friendliness. Si electrodes could enable miniaturization of energy-dependent devices by empowering the production of micro-batteries with high capacity. However, the use of Si on a commercial scale is quite less due to its low intrinsic electrical conductivity and huge volume expansion during the charge-discharge process with lithium ions^7,8^. This volume expansion of Si results in pulverization, and the structure of the prepared electrode is distorted by losing contact with the current collector (copper foil), which leads to drastic capacity loss within a few cycles. Rapid capacity fading and poor cycling are also attributed to continuous formation of SEI layer enabled by exposure to fresh Si particles^9^. To address these Si anode defects, various structures including nanospheres^10^, nanowires^11^, and nanotubes^12,13^ have been evaluated and several composites with either carbon^14,15^ or other materials that are inactive with lithium have been synthesized^16^.

Silicon with carbon as a composite or coating has been studied widely, because carbon can improve the mechanical flexibility of the active material and even after structural breakdown, it can provide robust electronic conductivity around the active material^17–19^. Several materials such as glucose, polyacrylonitrile, polyvinylalcohol, and coal tar pitch have been used as carbon sources. Kim *et al*. studied the Si/C anode involving aggregation of nanoparticles and showed excellent reversible capacity but the composite material was synthesized at high temperature and high pressure^20^. Tian H *et al*. reported the specific capacity of 1182 mAh g^−1^ at a current density of 50 mA g^−1^ for Si/C anode^21^. The benefits of Si/C anode gives long cycle life with superior rate capability and cycling stability. It was credited that the nano-Si with a carbon coating enhanced the electric conductivity and bonding between Si particles and the matrix. Even though there are several reports on Si/C anode, still there is a huge scope for research to obtain the full theoretical capacity^22^. In this article, we used biopolymer chitosan as a carbon source for the following reasons:

- Chitosan is the only alkaline polysaccharide in nature, rich in nitrogen^23^. The presence of nitrogen in the carbon structure is expected to enhance electron transport and electrochemical properties.
- Chitosan enables a facile and cost-effective synthesis of nitrogen-doped carbon (N-C) coated m-Si^23–25^.

Chitosan has been used extensively in biopharmaceutical and biomedical applications^26^. Chitosan has recently been used in Li-ion batteries for various purposes; in a previous study, we used chitosan as a binder for the cathode active material^27^. Chitosan has also been used as a template during the synthesis of active material, as a carbon source^28^, as a binder for several anode materials, and as a gel polymer electrolyte^29,30^.

Our objective in this study is to entrap silicon inside the nitrogen-doped carbon material by a facile method to control the volume expansion of silicon and to improve its conduction behavior. The physical properties of the N-C-entrapped Si was analyzed using X-ray diffraction (XRD), Raman spectrometer, thermogravimetric analysis (TGA), field emission transmission electron microscopy (FE-TEM), and X-ray photoelectron spectroscopy (XPS). Its electrochemical behavior during lithiation and delithiation (cycling) process was examined. In addition, we evaluated the formation and evolution of the SEI layer formed during the cycling process in Li-ion cells by XPS analysis.

## Results and Discussion

**Figure 1:**
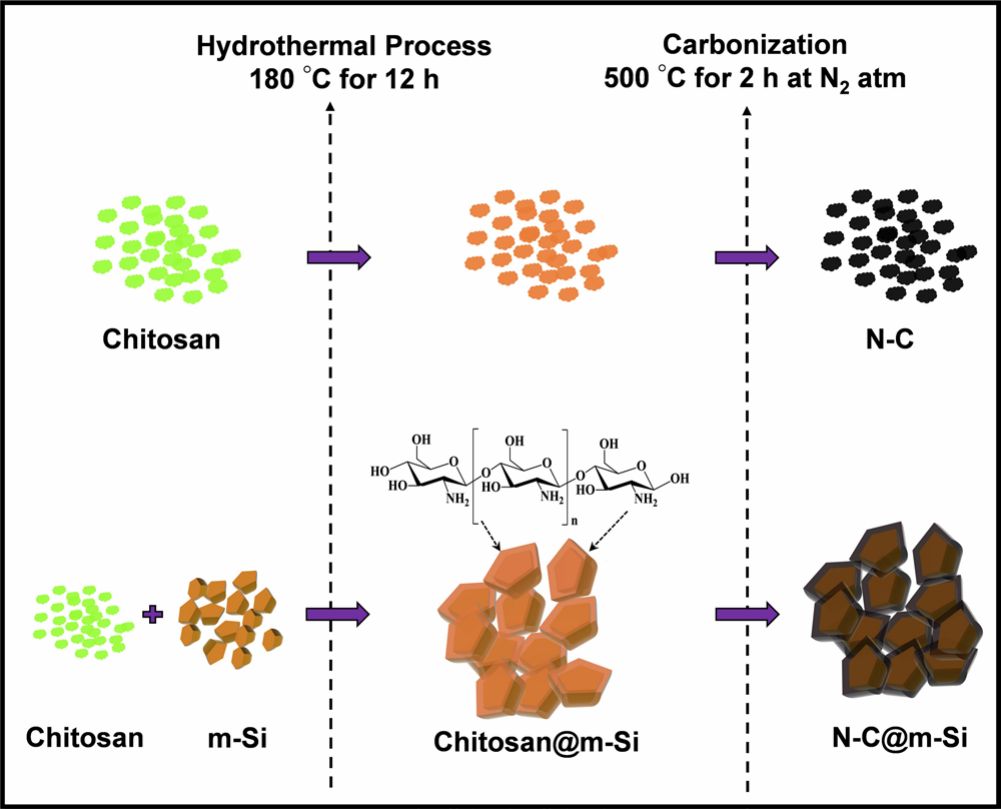
The preparation process of N-C and N-C@m-Si electrode materials.

Inter and intra molecular hydrogen bonding enables a rigid crystalline structure in chitosan. When chitosan and m-Si are mixed together in dilute acetic acid, they spontaneously form a compact nano complex with an overall positive surface charge. This property of chitosan leads to facile entrapment of m-Si nanoparticles to synthesis N-C@m-Si nanocomposites as shown in Fig. 1.

**Figure 2:**
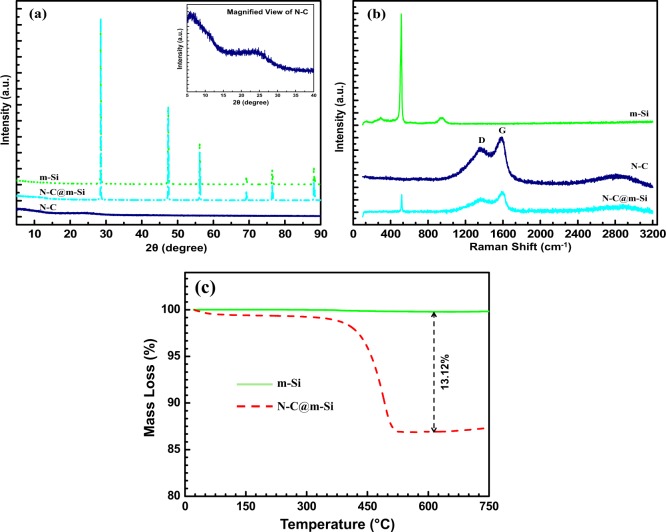
(**a**) XRD spectra, (**b**) Raman Spectra of m-Si, N-C@m-Si, and N-C samples and (**c**) TGA of as prepared m-Si and N-C@m-Si samples.

The XRD patterns of m-Si, N-C@m-Si, and N-C samples are shown in Fig. 2a. The peaks at 28°, 47°, 56°, 69°, and 76° was indexed as (111), (220), (311), (400), and (331) planes of Si crystals, respectively, forming a face-centered cubic lattice (JCPDS card no. 27-1402)^18^. The N-C@m-Si sample showed only Si peaks, confirming the absence of a phase change during the carbonization process at 500 °C in nitrogen atmosphere. Moreover, no diffraction lines of carbon was observed in the XRD pattern, thereby indicating that the residual carbon was amorphous in nature^19^.

We attributed an increased intensity of Si peaks in N-C@m-Si sample to the calcination process. The graphitic structure of N-C sample is exhibited by an observed broad peak, centered at 2θ = 26.5°. It corresponds to (002) plane according to the JCPDS card no. 89-8487. The presence of graphitic carbon in N-C@m-Si sample was confirmed by Raman analysis as shown in Fig. 2b. The peaks observed at 513.7 cm^−1^and 967 cm^−1^ corresponds to the crystalline nature of Si in m-Si sample. The graphitic structure of carbon observed in XRD is also confirmed by Raman spectra for N-C sample. The peak observed at 1347.8 cm^−1^ (D band) refers to the disordered carbon atoms, the peak at 1590.33 cm^−1^ (G band) refers to sp2 hybridized graphitic carbon atoms and the peak observed over the range 2600 cm^−1^ to 3090 cm^−1^ corresponds to the 2D band^23^. With regards to the N-C@m-Si sample, crystalline Si peak is observed at 517.7 cm^−1^ and the D and G band are observed at 1345.5 cm^−1^and 1584.18 cm^−1^, respectively^7^. The amount of carbon content in the N-C@m-Si sample was determined by TGA, and the results are shown in Fig. 2c. The slight weight loss occurring below 375 °C was due to the evaporation of water, observed on the surface of the sample. We attributed the dominant weight loss between 375 °C and 525 °C to the oxidation of nitrogen-doped carbon, which was about 13.12 wt%.

**Figure 3:**
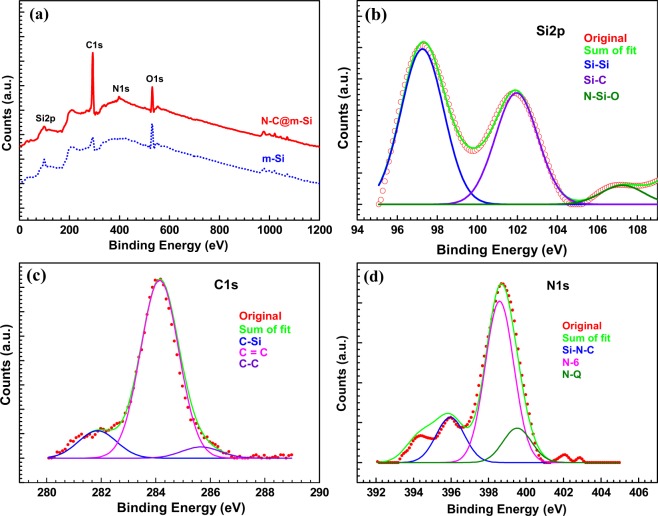
(**a**) XPS spectra of m-Si and N-C@m-Si samples. (**b**) Si2p, (**c**) C1s, and (**d**) N1s XPS spectrum of the N-C@m-Si sample.

The atomic configuration of N-C@m-Si was elucidated using XPS measurements as shown in Fig. 3. The interactions between silicon, carbon, and nitrogen are discussed in brief. The chemical interaction of silicon with carbon and nitrogen was determined by deconvoluting the Si2p peak (Fig. 3b) into Si-Si, Si-C, and N-Si-O peaks. In the C1s spectrum, peaks corresponding to C-Si, C=C, and C-C were observed, confirming the bonding between carbon and silicon as depicted in Fig. 3c. The interaction between carbon and silicon plays a major role in diffusion of ions during the electrochemical process in Li-ion batteries. The nitrogen N1s peak was deconvoluted into Si-N-C, N-6 (pyridine-like), and N-Q (graphite-like) peaks as shown in Fig. 3d; demonstrating the interaction of nitrogen with carbon and silicon. Interaction of nitrogen with silicon and carbon helps to improve the electronic conduction of an electrode. In other words, it helps in decreasing the polarization created during the electrochemical process and contributes to enhance the power and energy density^23,31,32^.

**Figure 4:**
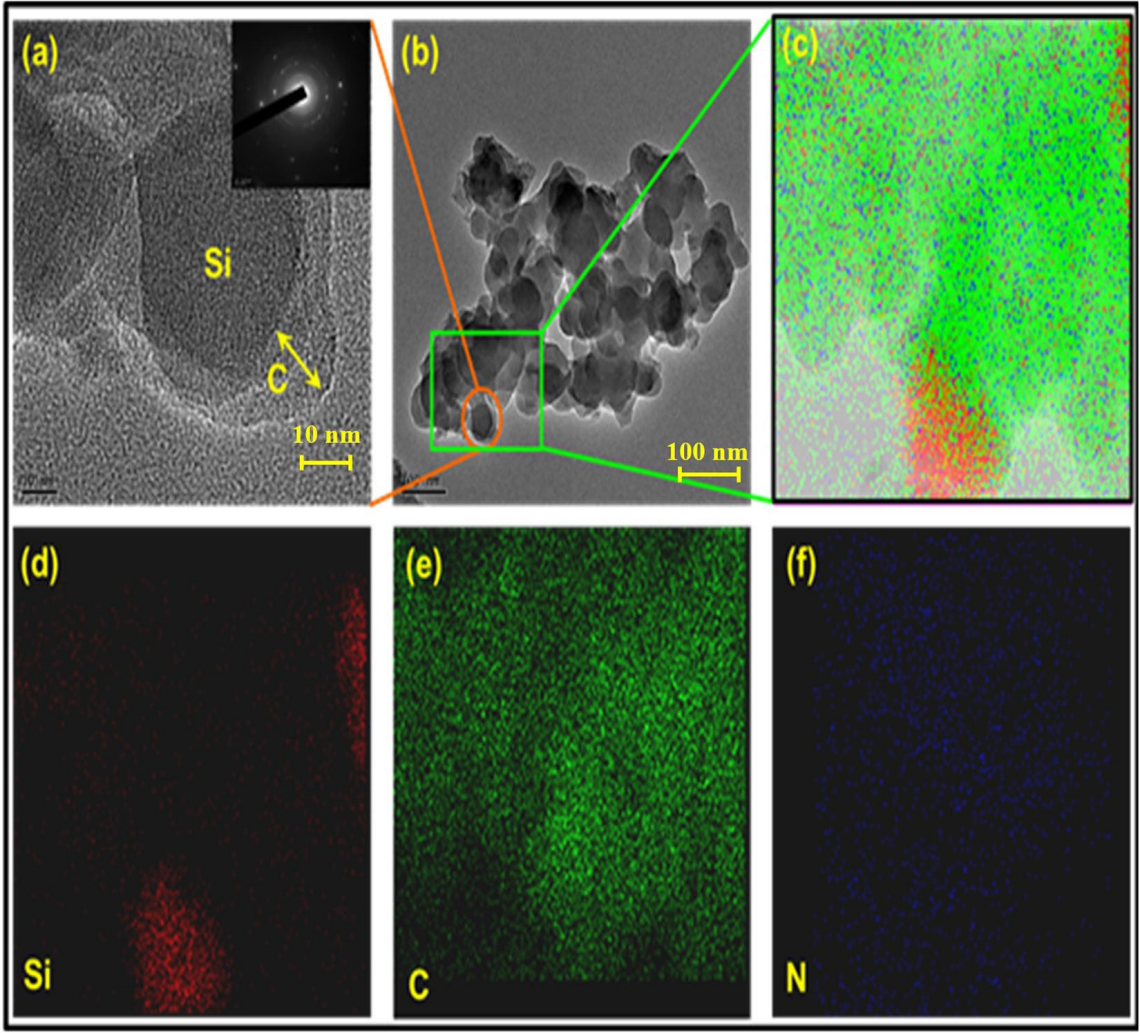
(**a**) FE-TEM images of the N-C@m-Si sample at high magnification of 10 nm. The inset shows the SAED pattern of specified location. (**b**) FE-TEM images of the N-C@m-Si sample at low magnification of 100 nm. (**c**) STEM images of the N-C@m-Si sample were obtained for elemental analyses. Elemental mapping of Si, C, and N are shown in (**d**–**f**), respectively.

The morphology of the synthesized N-C@m-Si sample was observed using FE-TEM. As shown in Fig. 4a, silicon was trapped in carbon matrix; in addition, carbon forms a strong interconnecting layer between silicon particles as shown in Fig. 4b^33^. The interconnecting layers are expected to improve the electrical contact between m-Si particles during the electrochemical processes. Figure 4a confirms the amorphous nature of carbon and allows estimation of thickness of the carbon layer over silicon (~10 nm). The selected area electron diffraction (SAED) pattern taken over the dark surface of N-C@m-Si sample (inset Fig. 4a) reveals several discontinuous diffraction rings corresponding to (111), (220), and (311) planes of silicon^25,34^. The scanning transmission electron microscopy analyses conducted over N-C@m-Si sample (Fig. 4c,f), shows an even distribution of carbon and nitrogen over silicon particles^35^.

**Figure 5:**
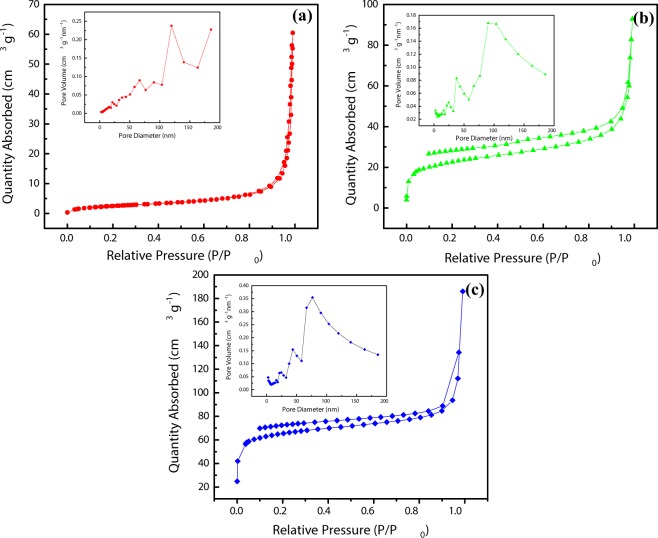
N_2_ adsorption/desorption isotherms of (**a**) m-Si, (**b**) N-C@m-Si, and (**c**) N-C samples. The inset images shows the corresponding pore size distributions.

The BET surface area and BJH pore size distribution of N-C and N-C@m-Si samples were measured as shown in Fig. 5a–c. The BET surface area of 80.72 m^2^ g^−1^ observed for N-C@m-Si sample, is comparatively smaller than the surface area of 241.99 m^2^ g^−1^ observed for N-C sample. The decrease in surface area also denotes a decrease in porosity, which is due to the insertion of m-Si nanoparticles into N-C. The m-Si sample has a very low surface area of 9.44 m^2^ g^−1^.

The N-C@m-Si sample shows higher surface area compared to the m-Si sample due to the presence of N-C layer on its surface. The obtained pore size distribution (BJH) of m-Si, N-C@m-Si, and N-C samples are ~119.52 nm, ~90.22 nm, and ~76.49 nm, respectively. The decrease in pore size of N-C@m-Si nanocomposites when compared with m-Si nanoparticles, also denotes the insertion m-Si in to the N-C particles^35^.

**Figure 6:**
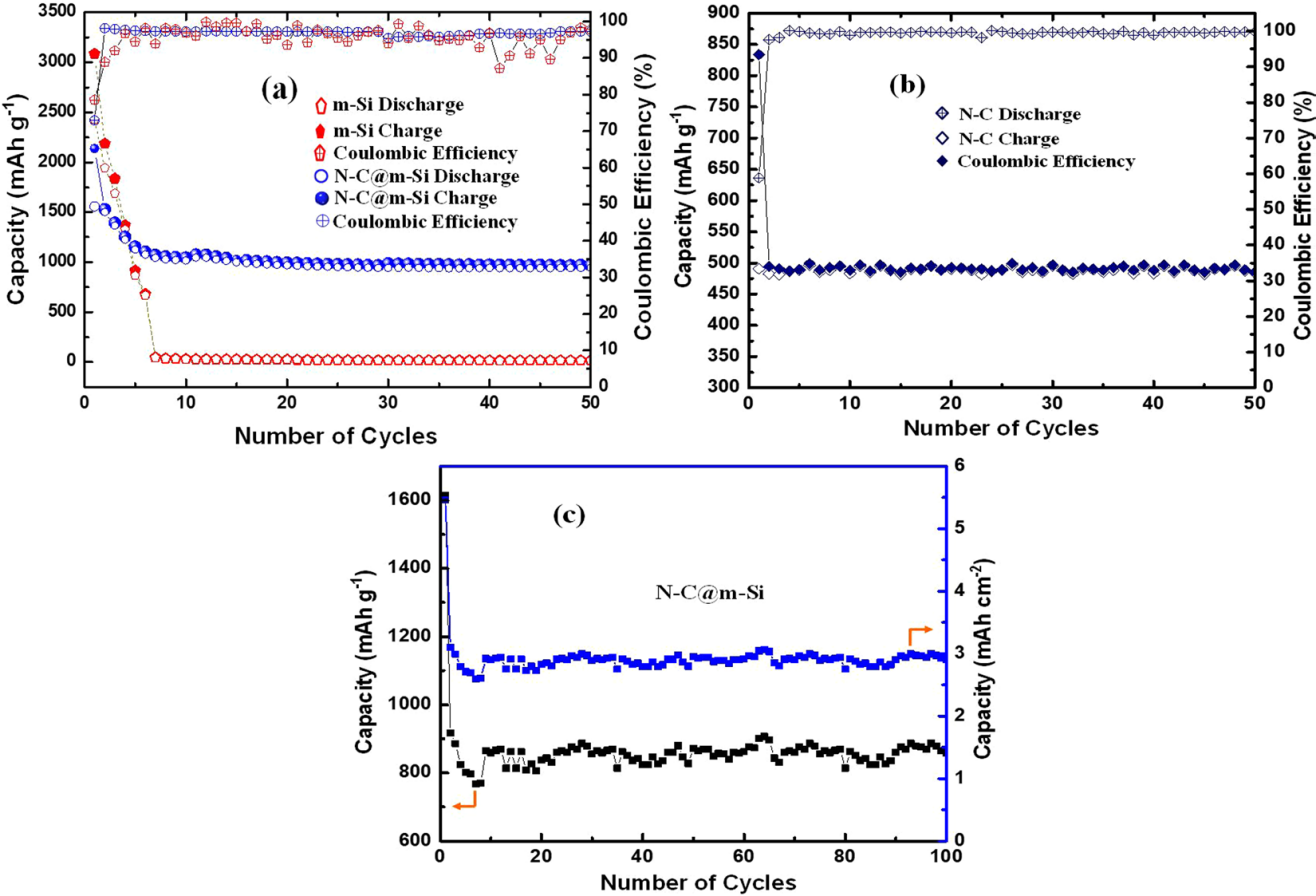
Specific capacity and columbic efficiency of (**a**) m-Si and N-G@m-Si, (**b**) N-G electrodes, and (**c**) Areal capacity and specific capacity for the N-G@m-Si electrode at 1C.

To examine the performance of m-Si, N-C, and N-C@m-Si electrodes in Li-ion batteries, prepared coin cells were discharged and charged (cycle) at a current rate of 0.1C between the voltage range of 0.01 V and 2 V. Coin cells with m-Si electrode had higher capacity and CE values on its first cycle, however these values rapidly decreased to zero after a few more cycles. Whereas the coin cells with N-C@m-Si electrode had lower capacity and CE values on its first cycle, but these values stabilized to a nominal capacity and good CE within a few cycles, as shown in Fig. 6a^36–38^ at slow C-rate. At 1C the N-C@m-Si electrode delivers a reversible capacity of ~852 mAh g^−1^ and areal discharge capacity of ~2.9 mAh cm^−2^ after 100 cycles as shown in Fig. 6(c). The obtained areal discharge capacity accentuates its energy storing ability for practical application. The electrode prepared for areal capacity measurement consists of 10% of PVDF binder, 10% of Super P, and 80% of active material N-C@m-Si. The improved electrochemical properties of N-C@m-Si electrode is due to the presence of carbonaceous substance with N-Q and N-6 functional groups. The quaternary N (N-Q) and pyridinic N (N-6) functional group enhances the electron transport between the electrodes and thereby increasing its capacity in Li-on batteries.

To stress the effect of nitrogen doping, the prepared N-C electrode is tested separately and compared with the well reported commercial graphite anodes. Figure 6b shows the electrochemical behavior of N-C electrode and it is comparatively better than the commercial graphite. This shows the importance of N-Q and N-6 functional groups in N-C and N-C@m-Si electrodes. The specific charge and discharge capacity were calculated using the total mass of the active material reported in experimental section.

**Figure 7:**
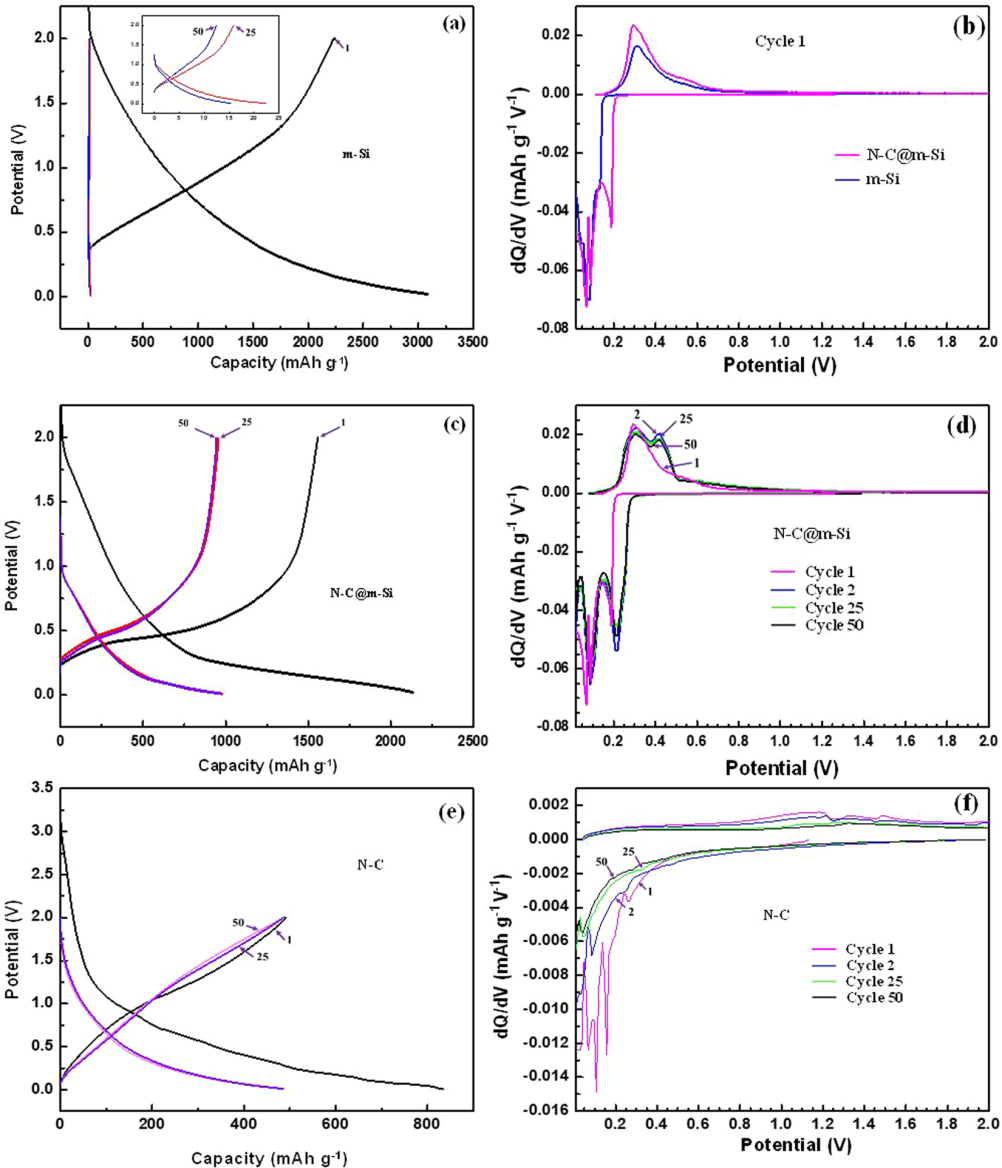
Charge-discharge plots of (**a**) m-Si, the inset image shows magnified views of the 25^th^ and 50^th^ cycles, (**c**) N-C@m-Si, and (**e**) N-C. (**b**) First cycle differential capacity curve of N-C@m-Si and m-Si electrodes. (**d**) Differential capacity curves of the N-C@m-Si electrode and (**f**) Differential capacity curves of the N-C electrode cycled between 0.01 V and 2.0 V at the C-rate of 0.1C.

To examine the cycling behavior of the samples in more detail, potential profiles for cycles at equal intervals were determined as shown in Fig. 7a,c, and e for m-Si, N-C@m-Si, and N-C electrodes, respectively. The charge-discharge capacity values of 1^st^, 25^th^, and 50^th^ cycles were reported for all the electrodes. The large irreversible capacity values obtained on the first cycle of m-Si, N-C, and N-C@m-Si samples are due to the formation of SEI layer and consumption of lithium ions. Due to high volume expansion behavior of silicon, m-Si particles are pulverized by the consumption of lithium ions, and these pulverized silicon particles interfered with the formation of SEI layer^39^. This resulted in an unstable SEI layer and rapid capacity fade, as shown in Fig. 7a. Coating of N-C, reduced the volume expansion behavior of silicon particles, resulting in the formation of a stable SEI layer^40^. In addition, high volume expansion behavior of silicon reduces the electronic conduction among the particles and between the particles and the current collector. The loss of electronic conduction induced a capacity drop with extended cycling.

**Figure 8:**
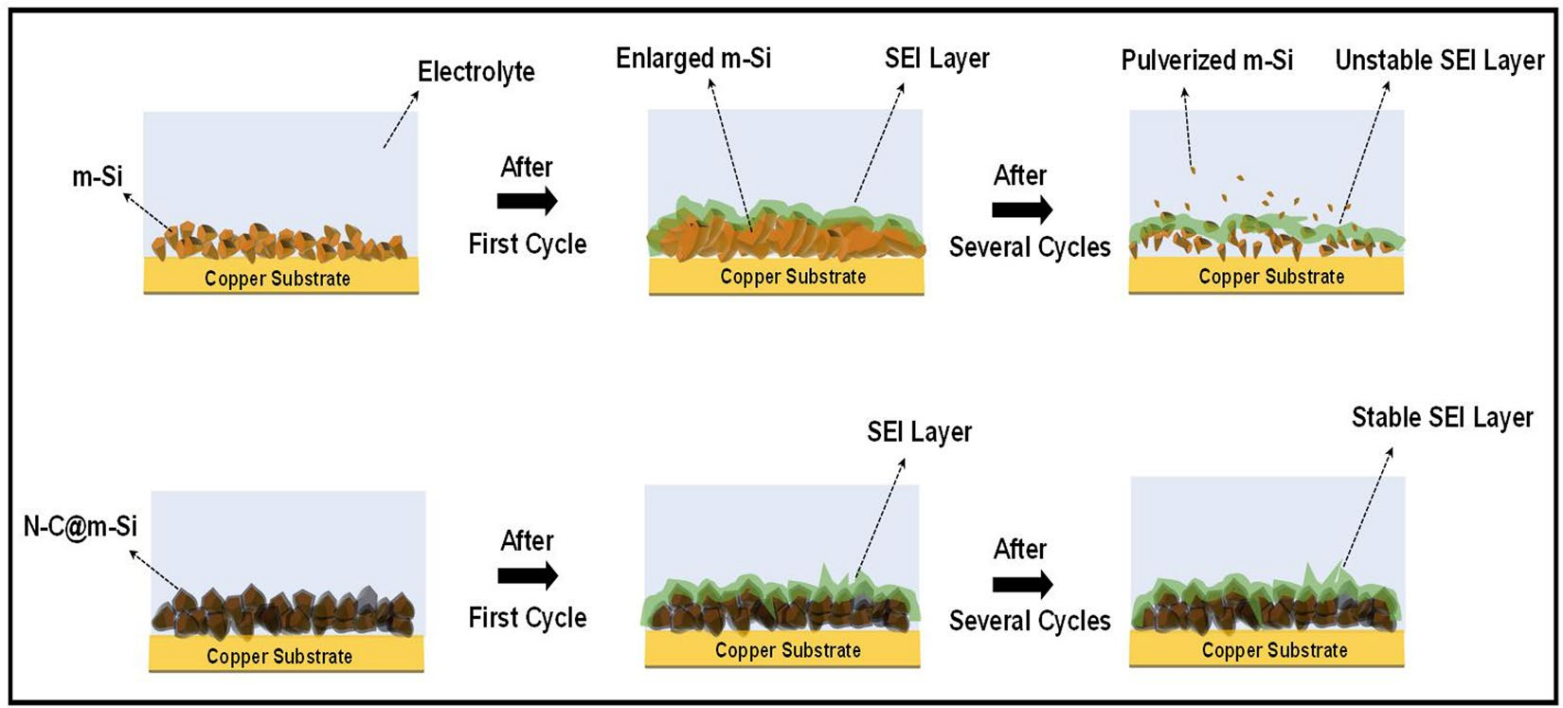
Formation of SEI layer on m-Si and N-C@m-Si electrodes with respect to cycle numbers.

The N-C@m-Si electrode shows significantly higher capacity and columbic efficiency due to a strong network among the particles and stable interactions between particles and the current collector. In addition, the N-C layer over m-Si increases electronic conductivities by generating excessive electrons. The presence of nitrogen enhances the connection between carbon and lithium ions, hence increasing the intercalation of lithium ions. The formation of SEI layer, connection between the particles and between the particle and current collector are pictorially represented in Fig. 8. With regards to the potential profile of N-C electrode (Fig. 7e), the first discharge curve shows small plateau at about 0.1 V, 0.25 V, and 0.6 V, due to the decomposition of electrolyte and formation of organic compounds. The observed plateaus disappear in subsequent cycles.

We compared the initial differential capacity profiles of m-Si and N-C@m-Si electrodes under ambient conditions as shown in Fig. 7b. The m-Si electrode had one reduction peak and one oxidation peak at 0.08 V and 0.28 V, respectively^38,41^. In contrast, the N-C@m-Si electrodes had two reduction peaks at 0.07 V and 0.22 V and one oxidation peak at 0.26 V. The oxidation and reduction peaks of N-C@m-Si electrode shifts to a lower voltage region as compared to the peaks for m-Si electrode. This shift of redox potential to a lower potential region is caused by different interactions between the electrode surface and the electrolyte, which results in varied kinetic responses. Both electrodes had a long plateau in the reduction region which is ascribed to the formation of a metastable amorphous phase of Li-Si formed as a result of solid-state amorphization reaction. We also compared the differential capacity profiles of N-C@m-Si electrode at different cycle intervals, namely the 1^st^, 2^nd^, 25^th^, and 50^th^ cycles, as shown in Fig. 7d. The corresponding redox peaks are observed for all cycles except the first cycle.

**Figure 9:**
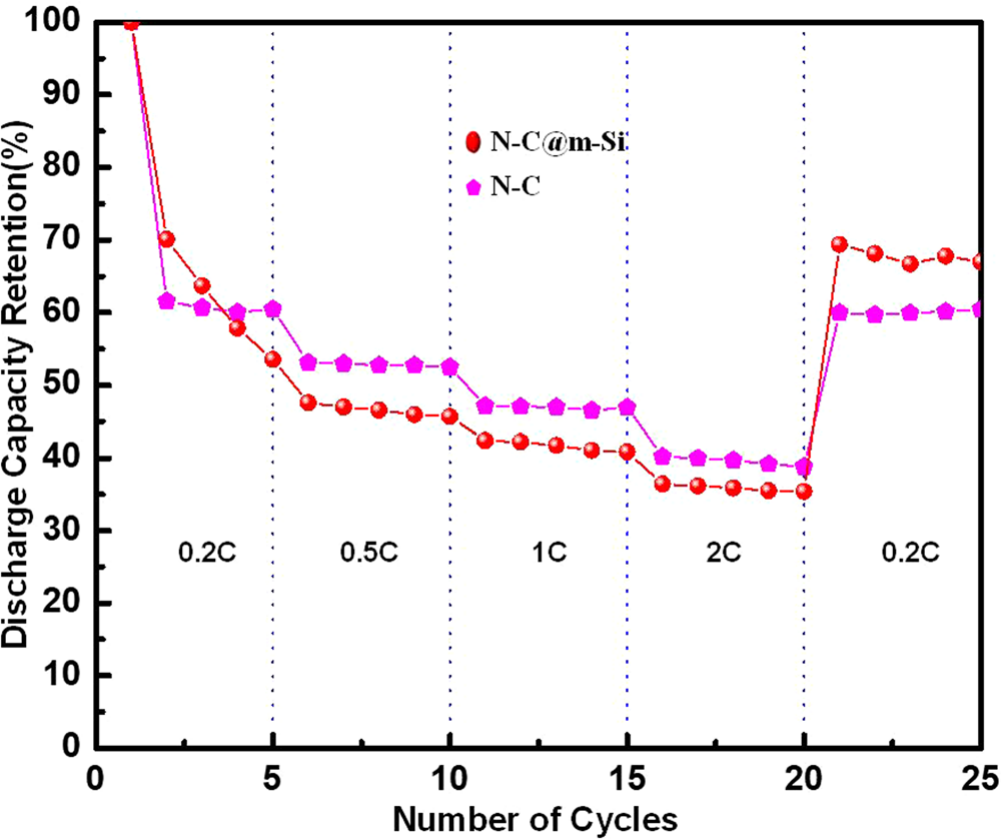
Discharge capacity retention percentage at different C rates.

We detected similar differential capacity profiles from the 2^nd^ cycle to the 50^th^ cycle. We ascribed the oxidation peak observed around 0.28 V to dealloying of Li from silicon, denoting the transition from PIII phase to PII phase. The oxidation peak at 0.44 V is attributed to the formation of amorphous silicon, and represents the transition from PII phase to PI phase. The reduction peak observed around 0.22 V corresponds to phase change from PI to PII phase, while the reduction peak around 0.07 V symbolized phase transition from PII phase to PIII phase^42,43^. Figure 7f shows the intercalation and deintercalation of Li ions into N-C electrode. The insertion of Li ions into N-C electrodes takes place at lower voltage close to 0.01 V, whereas the deintercalation occurs at about 0.85 V to 1.4 V. The small noise observed between 1 V and 1.5 V for first two charge curves are due to the extraction of Li-ions from oxygen functional groups residing on the surface of the carbonaceous material. This noise disappears with subsequent cycles due to the deactivation of the surface functional groups. Starting from the second cycle N-C electrode shows a very stable cycling behavior. The obtained positive characteristics of N-C could highly favor the m-Si upon coating. The N-C and N-C@m-Si electrodes also shows acceptable rate capability and high reversibility for lithium storage, as depicted in Fig. 9. Rate capability behavior of the electrodes is tested by varying the current density between 0.2C and 2C. During the second cycle, the N-C@m-Si electrode loses 30% percentage of its capacity obtained in the first cycle at 0.2C rate. But the capacity loss is comparatively less with subsequent cycling, even after increasing the C-rate. When the C-rate is brought back to 0.2C it retains about 70% of the obtained initial capacity. With regard to the N-C electrode it shows similar phenomena as explained for N-C@m-Si electrode and retains about 62% of the obtained initial capacity. The nitrogen in the carbon structure creates excess active sites and helps in adsorbing more lithium ions, resulting in enhanced Li-ion storage ability.

**Figure 10:**
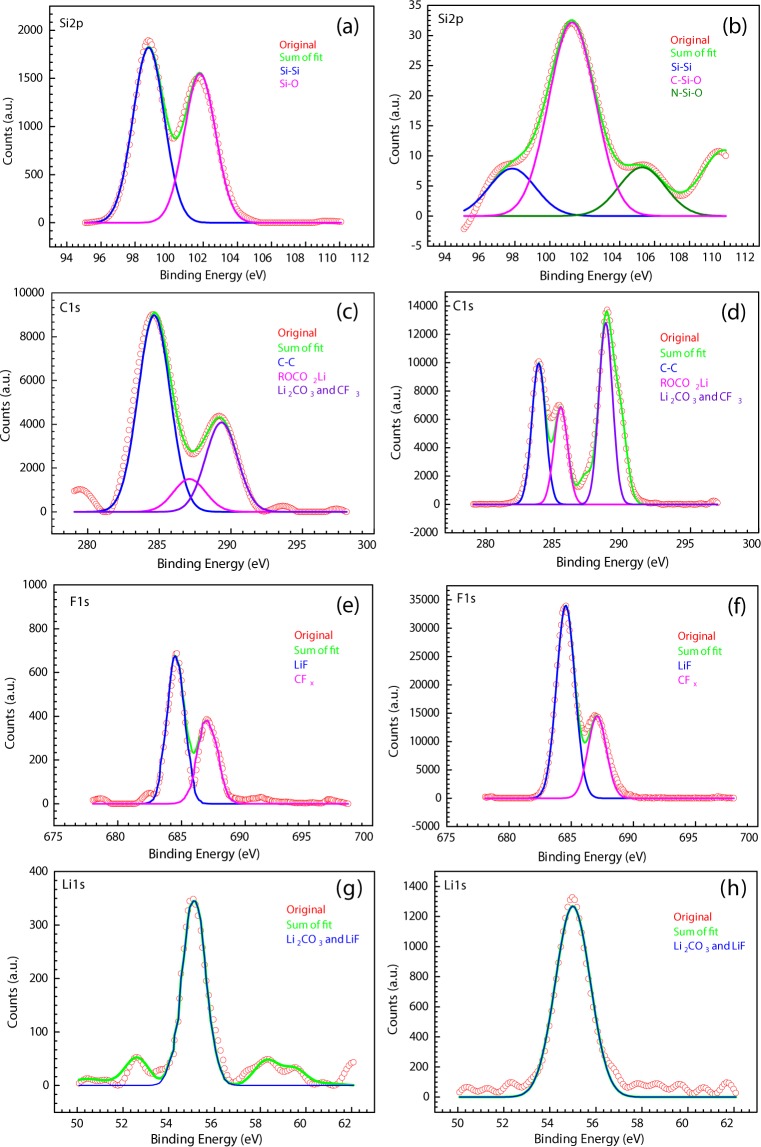
XPS spectra of m-Si electrode after 10 cycles: (**a**) Si2p, (**c**) C1s, (**e**) F1s, (**g**) Li1s. XPS spectra of N-C@m-Si electrode after 10 cycles: (**b**) Si2p, (**d**) C1s, (**f**) F1s, (**h**) Li1s.

To provide support for our hypothesis concerning the role of SEI layer during electrochemical cycling, the prepared coin cells were disassembled after 10 cycles in the fully charged condition to obtain the surface composition of electrodes using XPS analysis. Characteristic peak of Si obtained from m-Si and N-C@m-Si electrodes after 10 cycles is shown in Fig. 10a,b, respectively. A strong Si-O peak was observed for both electrodes, which was not expected. Si-O formation can occur during XPS analysis upon exposure of the cycled electrodes to the open atmosphere^44^. Several studies have reported the formation of an amorphous phase from crystalline Si after a few cycles. Amorphous Si is more prone to oxidation by air in an open atmosphere.

As observed in Fig. 10a, the peak intensity of Si in m-Si electrode was significantly higher than that present in N-C@m-Si electrode due to pulverization of Si during the lithiation and delithiation processes in m-Si electrode. This suggests that the pulverized Si particles lost contact with the current collector and moved to the upper surface of the electrode. Invasion of the SEI layer by these particles resulted in the formation of an unstable, weak SEI layer^45–47^. Hence, more Si was detected on the surface of the m-Si sample. On the surface of N-C@m-Si electrode (Fig. 10b), C-Si-O and Si-N elements were detected, confirming a complete coverage of Si particles by N-C, which aided in the formation of a stable SEI layer.

The C1s spectra of m-Si and N-C@m-Si electrodes shown in Fig. 10c,d confirms the presence of C-C, Li_2_CO_3_, CF_3_, and ROCO_2_Li compounds^48^. Decomposition of the electrolyte (EC/DEC) resulted in the formation of $C{O}_{3}^{2-}$, which reacted with lithium ion to form Li_2_CO_3_. The formation mechanism is shown below in eqs (1), (2), and (3).

In the case of EC:

1 $${(C{H}_{2}O)}_{2}CO+2{e}^{-}\to {C}_{2}{H}_{4}+C{O}_{3}^{2-}$$

In the case of DEC:

2 $${(C{H}_{3}C{H}_{2}O)}_{2}CO+2{e}^{-}\to {C}_{4}{H}_{10}+C{O}_{3}^{2-}$$

3 $$2L{i}^{+}+C{O}_{3}^{2-}\to L{i}_{2}C{O}_{3}$$

Further reduction of the electrolyte (EC/DEC) could result in the formation of alkyl carbonates.

In the case of EC:

4 $$2{(C{H}_{2}O)}_{2}CO+2{e}^{-}+2L{i}^{+}\to {C}_{2}{H}_{4}+2(C{H}_{2}OC{O}_{2}Li)$$

In the case of DEC:

5 $$2{(C{H}_{3}C{H}_{2}O)}_{2}CO+2{e}^{-}+2L{i}^{+}\to {C}_{4}{H}_{10}+2(C{H}_{3}C{H}_{2}OC{O}_{2}Li)$$

The C1s spectra of N-C@m-Si (Fig. 10d) electrode is highly stable and intense due to the presence of coated carbon layer over Si particles and also due to the formation of stable $C{O}_{3}^{2-}$ species^49^. The interaction of coated carbon with Si particle is also supported by the above observed Si2p spectra.

As shown in Fig. 10e,f, the F1s spectra of both samples had similar peaks for LiF, and CFx. But the intensity of the observed peaks is much lower for m-Si sample than N-C@m-Si sample. This difference in peak intensity is likely due to the differences in interaction between the electrolyte and the electrode. The intensity of observed LiF peaks signifies stability of the formed SEI layer. The formation of LiF can be initiated by two routes:

(i) Direct reaction of fluorophosphates species with lithium ions:

6 $$P{F}_{6}^{-}+2{e}^{-}+3L{i}^{+}\to 3LiF+P{F}_{3}$$

(ii) H_2_O contamination in the electrolyte, which can induce LiF formation as well as undesirable HF acid formation:

7 $${H}_{2}O+P{F}_{6}^{-}+L{i}^{+}\to LiF+PO{F}_{3}+2HF$$

SiF_6_ originated either by reaction of SiO_2_ with reduced fluorophosphates (Eq. 8) or by dissolution of SiO_2_ in HF acid (Eq. 9):

8 $$Si{O}_{2}+2P{F}_{6}^{2-}\to Si{F}_{6}^{2-}+2PO{F}_{3}$$

9 $$Si{O}_{2}+{H}^{+}+{F}^{-}\to Si{F}_{6}^{2-}+2PO{F}_{3}$$

The formation of fluorinated carbon species is caused by the reaction of polyethylene oxide with fluorinated Si^50^ (Eq. 10):

10 $${(-C{H}_{2}-C{H}_{2}O-)}_{x}+Si{F}_{6}^{2-}\to {(-C{F}_{2}-C{F}_{2}O-)}_{x}+Si{H}_{2x}{F}_{6-2x}^{2-}$$

Decomposition of fluorophosphates in the electrolyte leads to formation of LiF and PF_3_ compounds^51,52^. There is a possibility of fluorinated Si restoration by reaction with PF_3_ (Eq. 11):

11 $$P{F}_{3}+Si{H}_{y}{F}_{6-y}^{2-}\to P{H}_{y}{F}_{3-y}+Si{F}_{6}^{2-}$$

Li_2_CO_3_ and LiF peaks were also observed in Li1s spectra for both coated and pristine samples, as shown in Fig. 10g,h. The mechanism of formation of Li_2_CO_3_ and LiF is explained in Eqs 1–3 and Eqs 6, 7, respectively. The peak intensity of N-C@m-Si electrode is significantly higher than that of m-Si electrode due to the formation of a stable SEI layer. The correlation observed between Si2p, C1s, F1s, and Li1s spectra after 10 cycles of m-Si and N-C@m-Si electrodes strongly support the proposed hypothesis on the cycling behavior.

## Conclusions

We have developed a facile synthesis route for coating Si particles with N-doped carbon layers using biomaterial. The N-doped carbon layer derived from chitosan has played a key role in suppressing the volume expansion of Si particles and also in enhancing ionic and electronic conduction between the electrodes. The N-C@m-Si electrode has shown superior cycling performance as compared to bare m-Si electrode. The effect of nitrogen doping on carbon is compared with the reported bare graphitic materials. Cycling performance is interpreted by analyzing charge-discharge profiles and differential capacity profiles at equal cyclic intervals. To support our hypothesis on formation of SEI layer and to confirm its effect on cycling performance, the SEI layer was studied using XPS analysis. The N-doped carbon layer has facilitated the formation of a stable SEI layer over the surface of the electrode and also in maintaining the structural integrity of silicon during the lithiation-delithiation process. This in turn resulted in a stable cycling behavior. Using alkaline polysaccharide as a carbon source has enabled the development of a simple and effective methodology to address the drawbacks of silicon. This biopolymer can also be explored for use in other cathode and anode materials to enhance their electrochemical performance.

## Experimental

N-C and N-C@m-Si compounds were synthesized using a simple hydrothermal method followed by carbonization. Before the hydrothermal process, Si was ball milled for 24 h to reduce its size. In a typical hydrothermal process, pure chitosan and m-Si nanoparticles at 1:3 ratio was dispersed in 60 ml of distilled water containing 0.5 ml of acetic acid by gentle stirring. Acetic acid is used to dissolve the chitosan in distilled water. Gentle stirring was continued until a homogeneous solution was obtained. Chitosan was adsorbed over the surface of Si due to the inter-molecular hydrogen bonding. The obtained solution was transferred to a 100 ml Teflon-lined stainless steel autoclave, which was then sealed and maintained at 180 °C for 12 h. The obtained product was rinsed with water and ethanol several times and dried at 80 °C for 12 h. The hydrothermal process is followed by carbonization in nitrogen atmosphere at 500 °C for 2 h at a heating rate of 10 °C min^−1^. The N-C compound was synthesized using the above mentioned process without the addition of Si. Schematic representations of adsorption of chitosan on the surface of Si particles and further processes is provided in Scheme 1.

### Materials characterization

The structure of the m-Si, N-C, and N-C@m-Si nanoparticles was examined using XRD analysis in 2θ range between 5° and 90°. Raman spectroscopy was performed at the excitation wavelength of 457.9 nm. Thermogravimetric analysis (TGA) was carried to observe the presence of carbon and to measure its amount present in the synthesized samples at a heating rate of 10 °C min^−1^ under air. The compositions of the samples were investigated by XPS. XPS spectra is run on a K-alpha instrument (Thermo Electron) using monochromated Al Kα radiation (1486.6 eV) and a power of 350 W. The pressure in the chamber was 1.32*10^−9^ bar. Detailed scans were recorded for the Si2p, C1s, and N1s regions. The surface morphology of m-Si and N-C@m-Si were examined using FE-TEM. The specific surface area and pore size distributions were calculated using Brunauer-Emmett-Teller (BET) and Barrett-Joyner-Halenda (BJH), respectively.

For electrochemical analysis, anode electrodes were prepared by the doctor blade coating method using m-Si, N-C, and N-C@m-Si nano-powders as active materials, polyvinylidene difluoride (PVDF) as the binder, and Super P as the conductive agent at a ratio of 80:10:10 by mass and adding N-methyl pyrrolidone (NMP) to make a slurry. The prepared slurry was coated on thin copper foil and dried at room temperature for 24 h. The dried sample was then placed in a vacuum oven at 120 °C for 5 h. Lithium metal foil was used as the cathode electrode. 2032-type coin cells were assembled by sandwiching the separator (Celgard 2340) between the lithium metal and the prepared anode. One molar LiPF_6_ in ethylene carbonate: diethylene carbonate (EC: DEC, 1:1 vol%) was used as the electrolyte. The prepared 2032-type coin cells were cycled at room temperature at a constant current rate of 0.1C in a voltage range of 0.01 V and 2 V.

To demonstrate the formation and characterize the effects of the solid electrolyte interphase (SEI) layer, m-Si and N-C@m-Si anode electrodes were collected after the 10^th^ charge-discharge cycles. The collected samples were washed with DEC and vacuum-dried for 2 h. Dried samples were analyzed using an XPS instrument (K-Alpha, Thermo Electron).

### Ethical approval and informed consent

No live vertebrates (or higher invertebrates), humans or human samples were involved.

## Data Availability

All data generated or analysed during this study are included in this published article (and its Supplementary Information files).
